# Stepwise analysis of the scleral encircling learning curve for clinical fellows using surgical video review

**DOI:** 10.1186/s40942-026-00891-6

**Published:** 2026-06-26

**Authors:** Minjeong Kim, Yong Hoon Kim, Seok Hyeon Song, Yong Je Choi, Dong Ju Kim, Hyeong Min Kim, Chang Ki Yoon, Eun Kyoung Lee, Kihwang Lee, Un Chul Park, Se Joon Woo, Kyu Hyung Park, Seung Woo Choi

**Affiliations:** 1 Department of Ophthalmology, Seoul National University College of Medicine, Seoul National University Hospital, 101, Daehak-ro, Jongno-gu, Seoul 03080 Republic of Korea; 2 Department of Ophthalmology, Hangil Eye Hospital, Incheon, Republic of Korea; 3 Department of Ophthalmology, Seoul National University College of Medicine, Seoul National University Bundang Hospital, Seongnam, Republic of Korea; 4 Department of Ophthalmology, Konkuk University School of Medicine, Seoul, Republic of Korea

**Keywords:** Scleral encircling, Scleral buckling, Learning curve, Surgical education, Vitreoretinal fellowship, Indirect ophthalmoscopy

## Abstract

**Purpose:**

To identify which steps of the 360° scleral encircling (SE) procedure pose the greatest training challenges for retina fellows by analyzing surgical videos and evaluating changes in operative time across a two-year training period.

**Methods:**

We retrospectively analyzed videos of SE procedures performed by vitreoretinal fellows at a single academic center (2018–2024). Only cases involving 360° SE without concurrent vitrectomy or gas tamponade were included. For each case, total operative time and the duration of six predefined steps were measured. Cases with damaged videos or performed by fellows with ≤ 5 analyzable cases were excluded. Between-fellow comparisons and learning-curve trends were assessed using analysis of variance and correlation analyses.

**Results:**

Fifty-five SE cases performed by six fellows met inclusion criteria. Mean total operative time was 132.27 ± 42.92 min, with significant inter-individual differences (*p* = 0.0007). Among all steps, binocular indirect ophthalmoscopy showed the greatest inter-individual variability (*p* < 0.0001), followed by scleral suturing (*p* = 0.0009) and tire positioning (*p* = 0.006). Across training, the duration of indirect ophthalmoscopy did not show measurable reduction for any fellow. Five of six fellows demonstrated a significant decrease in total operative time correlated with training progression (*p* < 0.05 for each), with a marked early decrease within their first five cases, followed by a plateau.

**Conclusion:**

Binocular indirect ophthalmoscopy demonstrated the largest variability and the least improvement during SE training. Prioritizing targeted instruction in this technically demanding step may accelerate skill acquisition and improve overall operative efficiency for early-career ophthalmologists.

## Introduction

 Scleral buckling (SB), scleral encircling (SE) and pars plana vitrectomy (PPV) are established surgical techniques for the treatment of rhegmatogenous retinal detachment. Compared to PPV, SB offers several advantages including avoidance of intraocular manipulation, lower risk of cataract formation, faster visual recovery, and no requirement for postoperative face-down positioning. These benefits are particularly notable in younger phakic patients [1–7]. It also eliminates the risk of iatrogenic retinal breaks, causes less retinal displacement [6], and is more cost-effective [7]. In addition, combining SB with PPV has been shown to improve single-surgery anatomic success rates, particularly in cases involving inferior retinal breaks [8].

Despite these advantages, the use of SB has declined with the advancement of minimally invasive PPV techniques [9, 10]. This trend may be partly attributable to the technical challenges inherent to SB, which relies on binocular indirect ophthalmoscopy and scleral indentation—skills that can pose a steep learning curve for trainees more accustomed to slit-lamp-based ophthalmoscopy. The difficulty in sharing the surgical field between the surgeon and assistant further complicates its use as a teaching tool [8].

Notably, SB outcomes have been reported to improve significantly with higher annual case volumes [11] and approximately 30 cases are considered necessary to achieve stable clinical results [12]. However, attaining adequate SB exposure during fellowship training remains challenging, a problem further compounded by the COVID-19 pandemic [13].

Both SE and SB share core surgical steps as binocular indirect ophthalmoscopy and scleral indentation, which pose similar technical challenges for surgeons in training [14]. Because 360-degree SE requires consistent application of these skills while minimizing procedural variability inherent in segmental buckling, it may provide a relatively standardized model for evaluating the SB learning curve.

The SE procedure was divided into distinct steps, and surgical videos from procedures performed throughout a two-year retina fellowship program were retrospectively analyzed to assess the time required for each step and identify which components pose the greatest training challenges.

## Methods

This retrospective observational study was conducted in accordance with the tenets of the Declaration of Helsinki and was approved by the Institutional Review Boards of Seoul National University Hospital (IRB No. H-2507-129-1659) and Seoul National University Bundang Hospital (IRB No. B-2510-1005-401). The study utilized data from Seoul National University Bundang Hospital and was carried out at Seoul National University Hospital, the main institution. IRB approval was obtained from both institutions. The requirement for written informed consent was waived owing to the retrospective nature of the study and the use of anonymized patient data.

SE procedures performed by vitreoretinal fellows between March 2018 and February 2024 at Seoul National University Bundang Hospital were retrospectively reviewed. Cases were excluded if gas injection or vitrectomy was performed concurrently with SE. All surgeries were video recorded; cases with damaged or missing video files were classified as unanalyzable. Fellows with five or fewer analyzable cases were excluded from the final analysis. All fellows completed a two-year retina fellowship, and all SE procedures were performed under general anesthesia.

Detailed characterization of retinal break type and complexity was not feasible in all cases, as 360-degree SE is often indicated in eyes with multiple or poorly demarcated breaks where segmental buckling would be insufficient [15–17].

**Fig. 1:**
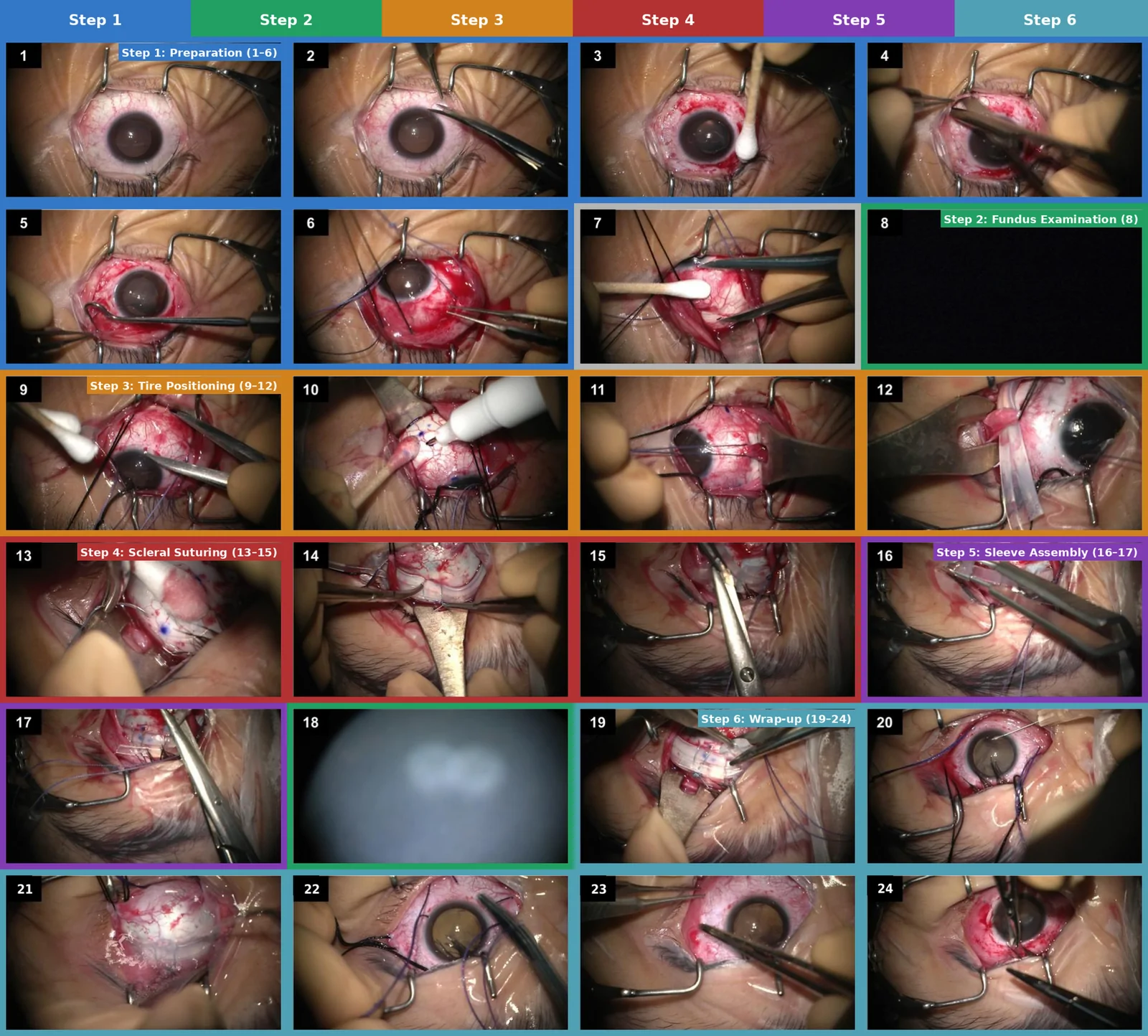
Surgical steps of scleral encircling, grouped according to the six predefined procedural steps **Step 1 – Preparation (1–6)**: A speculum was positioned at the beginning of the surgery (1). A 360-degree conjunctival peritomy was performed (2–3), followed by isolation of the extraocular muscles and placement of traction sutures for each muscle (4–6). **Step 2 – Fundus examination (8)**: After measuring the estimated distance of the retinal tears from the limbus (7), the first examination using an indirect ophthalmoscope and cryotherapy were performed (8). **Step 3 – Tire positioning (9–12)**: The ideal location for the tire was marked in all four quadrants (9–10), and the tire was placed beneath the four rectus muscles (11–12). **Step 4 – Scleral suturing (13–15)**: Scleral sutures were placed in all four quadrants to secure the tire and band (13–15). **Step 5 – Sleeve assembly (16–17)**: Both ends of the band were inserted into the sleeve and tightened (16), and the tire was trimmed to an appropriate length to achieve the desired indentation effect (17). **Step 6 – Wrap-up (19–24)**: A second indirect ophthalmoscopic examination was performed (18, the duration of the fundus examination step was calculated as the combined time of images 8 and 18.), followed by readjustment of the tire (19) and anterior chamber paracentesis if needed (20). To conclude the procedure, the eye was irrigated with a gentamicin solution (21), traction sutures were removed (22), and the conjunctiva was closed with sutures (23–24).

For each video, the total surgical time (from first incision to conjunctival closure) was recorded. Additionally, the durations of the following steps were measured:

1. Preparation: 360-degree conjunctival peritomy, isolation of extraocular muscles, and placement of traction sutures on all four rectus muscles.
2. Fundus examination: Observation of the retina using binocular indirect ophthalmoscopy. The duration of cryotherapy was included within the fundus examination step, as these maneuvers were performed sequentially under indirect ophthalmoscopic visualization.
3. Tire positioning: Placement of the tire and band beneath all four rectus muscles.
4. Scleral suturing: Average time required to place scleral mattress sutures to securely fix tire and band to the sclera.
5. Sleeve assembly: Insertion of both ends of the band into the sleeve and tightening.
6. Wrap-up: From antibiotic irrigation to conjunctival closure (Fig. 1).

Stepwise durations were analyzed for individual variability and learning trends. Statistical analysis was performed using SPSS software version 20.0 (SPSS Inc., Chicago, IL, USA) and Prism version 8.4.3 (GraphPad Software, La Jolla, CA, USA). Depending on data distribution and the number of groups, comparisons were made using one-way ANOVA or the Kruskal–Wallis test. Pearson’s correlation analysis was used to assess the relationship between surgical step duration and fellowship progression. A p-value of < 0.05 was considered statistically significant.

## Results

Over the six-year study period, a total of 123 SEs were performed by fifteen fellows. Nine fellows were excluded from the study due to having five or fewer analyzable surgical videos. As a result, 55 SE cases performed by six fellows were included in the analysis. Each fellow is anonymized and referred to as F1 through F6 (Table 1**).** All six followed a consistent surgical sequence (Fig. 1).

**Table 1:** Overall number of encircling cases of fifteen vitreoretinal fellows during their training period

No.		Total no. of videos	No. of analyzable videos	Inclusion	Fundus exam before encircling
1	Fellow A	3	3		
2	Fellow B	2	2		
3	Fellow C	8	5		
4	Fellow D	3	3		
5	Fellow E	9	7	Included (F1)	Done
6	Fellow F	3	3		
7	Fellow G	13	12	Included (F2)	Skipped
8	Fellow H	4	4		
9	Fellow I	5	5		
10	Fellow J	4	4		
11	Fellow K	24	13	Included (F3)	Done
12	Fellow L	12	8	Included (F4)	Done
13	Fellow M	2	2		
14	Fellow N	12	8	Included (F5)	Done
15	Fellow O	11	7	Included (F6)	Skipped
	Total	123	86		

Two fellows (F2 and F6) performed binocular indirect ophthalmoscopy both before and after sleeve tightening during their early training. As their experience progressed, they omitted the initial examination and performed only a single assessment after buckle assembly. This change appeared to reflect increased confidence in localizing retinal breaks and assessing buckle height intraoperatively, rather than a systematic change in institutional protocol or case selection. Their data were excluded from analyses involving fundus examination duration (Table 1).

The average age of the 55 patients was 36.1 ± 16.2 years, and 21.8% were female (Table 2). There were no statistically significant differences among the fellows in terms of the patient age, sex distribution, or the rate of reoperation within two months. Based on video analysis, the mean total surgical time for a single SE procedure was 132.27 ± 42.92 min (Table 3). The mean durations of individual surgical steps were as follows: 14.61 ± 3.78 min for preparation, 18.74 ± 11.45 min for fundus examination with an indirect ophthalmoscope, 6.47 ± 4.49 min for tire positioning, 6.92 ± 2.65 min for scleral suturing, 3.12 ± 2.09 min for sleeve assembly, and 16.26 ± 4.34 min for the wrap-up step. Total surgical time showed statistically significant inter-individual differences. Among the steps, the greatest variation was observed in fundus examination, followed by scleral suturing and tire positioning. No significant differences were found in preparation, sleeve assembly, or wrap-up durations.

**Table 2:** Demographics of patients who received encircling surgery by six vitreoretinal fellows

	Total	F1	F2	F3	F4	F5	F6	*p*-value
Total No. of cases	81	9	13	24	12	12	11	
No. of included cases	55	7	12	13	8	8	7	
Age (included cases)	36.05 ± 16.21	33.86 ± 8.07	34.33 ± 14.53	27.77 ± 13.16	44.25 ± 18.87	38.57 ± 16.62	40.14 ± 22.55	0.1960
Female (included cases)	12 (21.8%)	3 (42.9%)	3 (25.0%)	0	3 (37.5%)	3 (37.5%)	0	0.1787
Reoperation within 2 months (included cases)	11 (20.0%)	1 (14.3%)	0	4 (30.8%)	2 (25%)	1 (12.5%)	1 (14.3%)	0.4682

**Table 3:** Average time for total surgery and each step – individual variation

	Total	F1	F2	F3	F4	F5	F6	*p*-value
No. of surgery	55	7	12	13	8	8	7	
Total surgery time (min)	132.27 ± 42.92	113.43 ± 21.48	121.17 ± 14.81	130.31 ± 46.29	191.13 ± 62.51	116.25 ± 26.91	124.86 ± 18.29	**0.0007**
Preparation (min)	14.61 ± 3.78	14.24 ± 2.87	13.92 ± 2.12	15.75 ± 4.32	13.66 ± 4.10	13.19 ± 4.88	16.72 ± 3.83	0.3673
Indirect ophthalmoscope (min)	18.74 ± 11.45	12.02 ± 6.57	20.86 ± 9.97	16.00 ± 4.81	31.73 ± 12.81	12.13 ± 2.36	19.66 ± 18.67	**< 0.0001**
Tire positioning (min)	6.47 ± 4.49	6.72 ± 2.85	3.60 ± 2.16	6.97 ± 5.36	5.53 ± 2.47	10.34 ± 6.85	6.88 ± 2.63	**0.006**
Scleral suture (min)	6.92 ± 2.65	5.74 ± 0.68	5.28 ± 0.75	7.18 ± 3.53	10.30 ± 2.53	6.09 ± 1.35	7.50 ± 1.90	**0.0009**
Sleeve assembly (min)	3.12 ± 2.09	3.64 ± 1.83	3.49 ± 2.34	2.34 ± 0.86	3.94 ± 3.79	2.27 ± 0.93	3.44 ± 1.60	0.3296
Closing (min)	16.26 ± 4.34	14.38 ± 3.78	15.42 ± 4.02	17.06 ± 5.23	17.64 ± 5.28	17.59 ± 3.66	15.04 ± 3.06	0.5224

Table 4 summarizes the learning curve for each fellow by comparing the duration of surgical steps over time. For five of the six fellows, total surgical time decreased significantly as training progressed. Fellow F4, however, showed no statistically significant change in overall surgical time (*p* = 0.2923). While the specific steps showing time reduction varied among fellows, fundus examination remained unchanged across all individuals (excluding F2 and F6, who later omitted the first examination). Compared to peers, F4 showed prolonged durations in both fundus examination and scleral suturing (Table 5).

**Table 4:** Correlation analysis between the days of training period and average time for total surgery and each step

*P*-value	F1	F2	F3	F4	F5	F6
No. of surgery	7	12	13	8	8	7
Total surgery time (min)	**0.0370**	**0.0237**	**0.0116**	0.2923	**0.0196**	**0.0167**
Preparation (min)	**0.0254**	0.2718	**0.0241**	0.1508	**0.0309**	0.0607
Indirect ophthalmoscope (min)	0.1092	**0.0054**	0.1733	0.9408	0.4975	**0.0486**
Tire positioning (min)	0.0705	0.2124	**0.0364**	0.1595	**0.0106**	0.2623
Scleral suture (min)	0.3722	0.7723	**0.0411**	0.4861	0.2285	**0.0092**
Sleeve assembly (min)	0.3267	0.0807	0.0848	0.1030	0.1149	0.8293
Closing (min)	0.4106	0.9479	**0.0213**	0.1929	0.2635	0.5364

**Table 5:** Comparison between F4 and the other five vitreoretinal fellows

	F4	Others (*n* = 5)	*p*-value
No. of surgery	8	47	
Total surgery time (min)	191.13 ± 62.51	122.26 ± 29.38	**0.0002**
Preparation (min)	13.66 ± 4.10	14.77 ± 3.75	0.4508
Indirect ophthalmoscope (min)	31.73 ± 12.81	16.53 ± 9.73	**< 0.0001**
Tire positioning (min)	5.53 ± 2.47	6.63 ± 4.75	0.7381
Scleral suture (min)	10.30 ± 2.53	6.34 ± 2.23	**< 0.0001**
Sleeve assembly (min)	3.94 ± 3.79	2.98 ± 1.67	0.8745
Closing (min)	17.64 ± 5.28	16.03 ± 4.19	0.3375

**Fig. 2:**
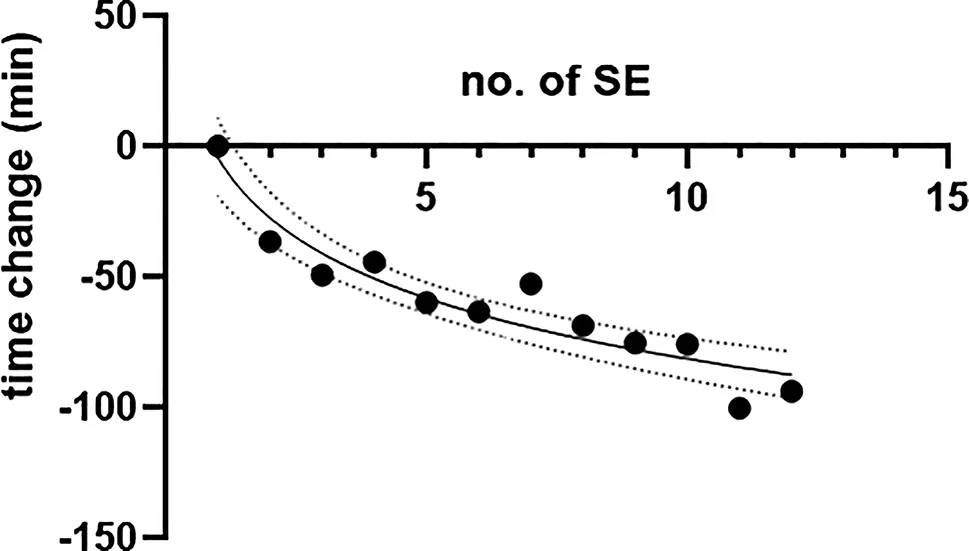
Change in total surgery time according to the number of experiences (five fellows, F4 excluded) *SE: Scleral encircling. Dotted line indicating the range of 95% confidence interval. Semilog line, least squares fit. Y=10^(-77.31*X-4.180)

**Fig. 3:**
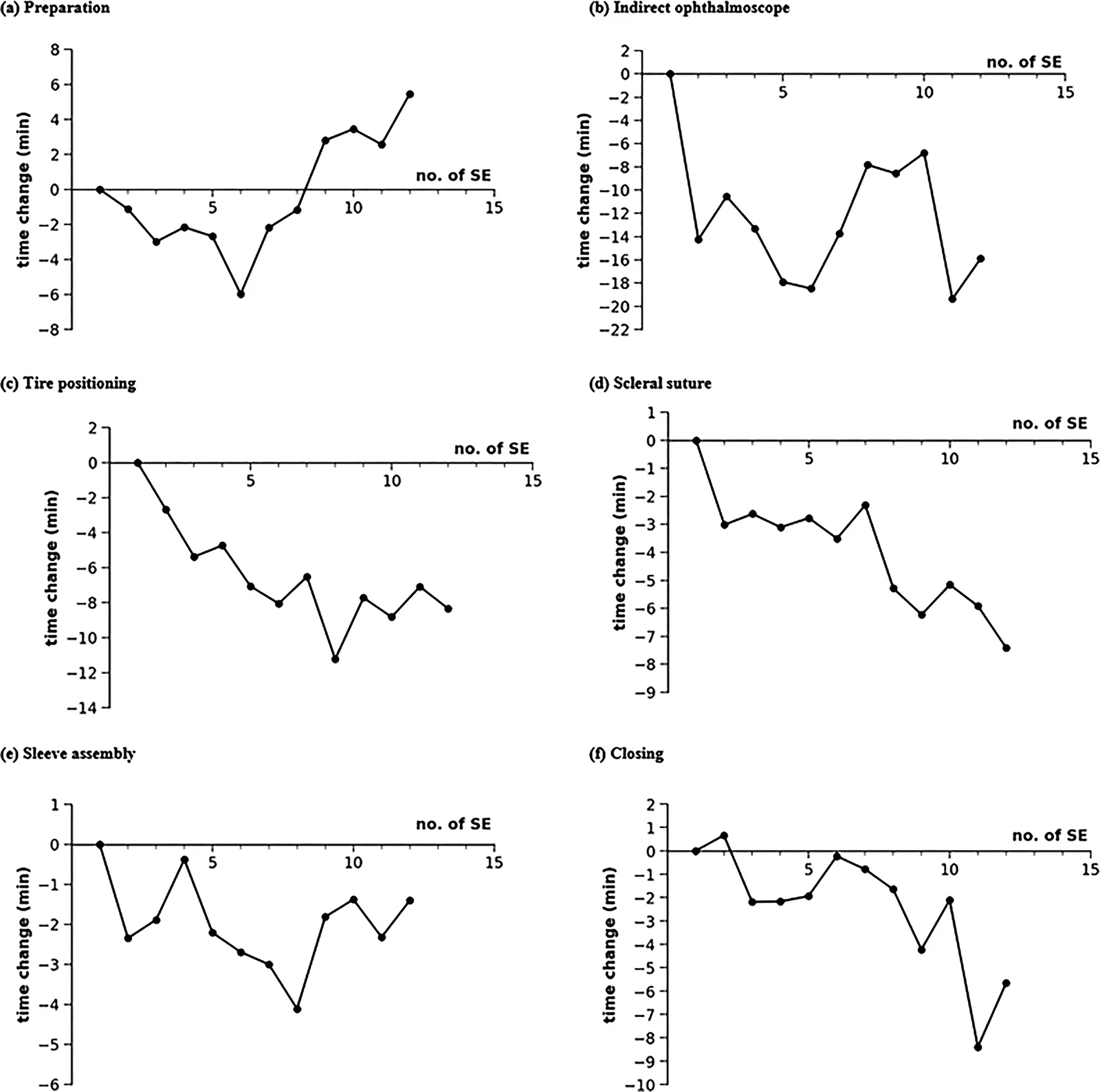
Time changes of each step according to the number of experiences (six fellows, F4 included) *SE: Scleral encircling

**Fig. 4:**
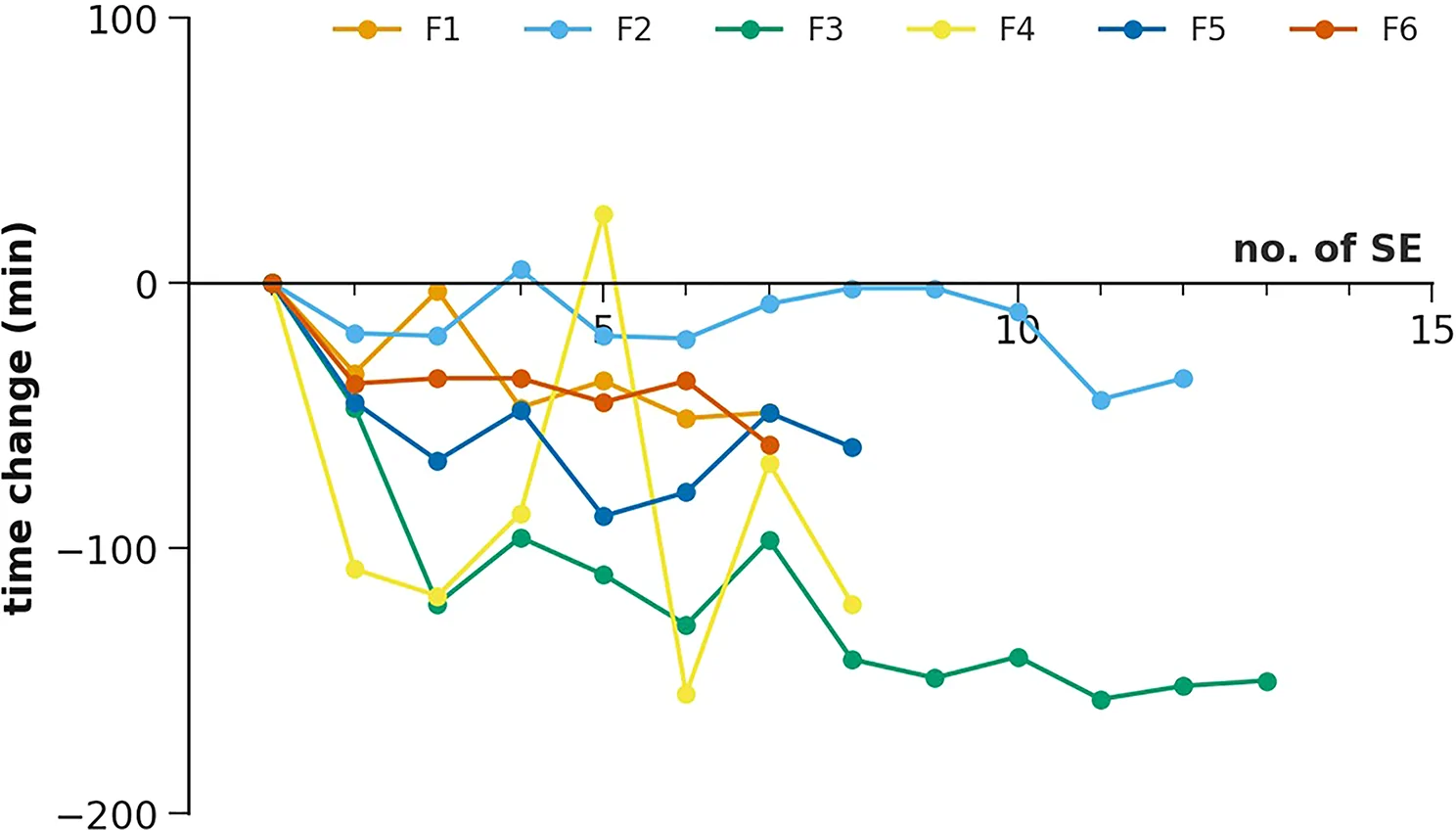
Individual change in total surgery time according to the number of experiences (six fellows, F4 included) *SE: Scleral encircling

Figure 2 shows the reduction in operative time across successive SE cases for each fellow (excluding F4). A marked decline in surgical duration was observed over the first five cases, with a plateauing trend thereafter. By the tenth case, the mean operative time had decreased by 76 min compared to the first surgery. Time changes of each step and individual change in total surgery time according to the number of experiences is demonstrated in Figs. 3 and 4.

## Discussion

This study analyzed 55 SE procedures performed by six vitreoretinal fellows during their two-year training period and found that the average surgical time was approximately 132 min—longer than durations reported in previous studies. One prior study evaluating the first 32 scleral buckling cases by a single surgeon reported an average operative time of 106 min; however, that analysis included segmental buckling procedures, whereas the present study focused exclusively on 360-degree SE [12]. Another report, which did not specify the surgeon’s level of experience, noted an average time of 105 min for encircling procedures, 27 min shorter than our findings [18]. This discrepancy is likely attributable to the experience of surgeons. Supporting this interpretation, a recent study on scleral buckle operative times at an academic center attributed relatively prolonged durations in part to greater case complexity and trainee involvement inherent to such settings [19]. Consistent with this, trainees have been reported to require more than twice the operative time of experienced surgeons for retinal detachment procedures [2].

By analyzing the average duration of each surgical step, this study provides insight into which components consume the most time and may benefit from targeted instruction. Marked inter-individual variability was noted in certain steps, particularly fundus examination with an indirect ophthalmoscope—likely reflecting fellows’ limited prior experience with binocular indirect ophthalmoscopy compared to slit-lamp-based method [13]. Importantly, this step showed no significant reduction in duration across the training period for any fellow, except for F2 and F6, who omitted the initial examination as their technique evolved. The fellow who showed no reduction in total surgical time over the course of training also required more time for indirect ophthalmoscopy than the other fellows.

This finding suggests that focused training in binocular indirect ophthalmoscopy is warranted. To improve proficiency, vitreoretinal fellowship programs may consider increasing hands-on exposure to the device. At our institution, fellows were trained through supervised operating room exposure, stepwise participation in live surgery, and progressive autonomy under faculty oversight. In addition to this, instructors could use chandelier illumination during surgeries and share their view or let trainees practice using the binocular indirect ophthalmoscope to perform indentation, observe the fundus, and confirm the appropriate height of the buckle [20, 21].

For scleral suturing, which exhibited the second highest inter-individual variability following indirect ophthalmoscopy and posed difficulty for the fellow who failed to reduce surgical time, real-time guidance on suture depth and optimal placement relative to extraocular muscles and vortex veins may be particularly valuable. The sleeve assembly step did not demonstrate a decrease in time with increased surgical experience, which is likely due to its relatively brief duration of approximately 2 to 3 min.

Consistent with previous reports suggesting a learning curve in scleral buckling [12], our results demonstrated that surgical duration declined steeply during the first five cases, with a slower improvement trend thereafter (Fig. 2). This supports the notion that early, targeted instruction—especially before trainees begin performing independent cases—can mitigate the steepness of the initial learning curve.

This study has several limitations. First, it was conducted at a single center, which may limit generalizability. Moreover, at our institution, SE is performed with a silicone tire in addition to a band, whereas some centers only use band [22]. Differences in buckling elements and surgical configuration may influence operative time and technical demands, which should be considered when extrapolating these findings to other settings. Second, surgical duration alone does not fully reflect procedural quality or surgical competency. Third, although stepwise durations were analyzed individually, a multivariate approach incorporating fellow experience level, case complexity, break characteristics, and preoperative planning strategy may have provided more robust conclusions. Due to the retrospective design and limited sample size, such analysis was not feasible and should be considered in future prospective studies. Fourth, detailed documentation of retinal break type and complexity was not consistently available across all cases. This is inherent to the nature of SE, which is preferentially indicated in eyes with multiple, poorly localized, or indistinct breaks for which segmental buckling is deemed insufficient. As such, the heterogeneity in break characteristics could not be fully accounted for in the analysis [15–17]. Finally, the analysis was limited to cases with analyzable surgical video, potentially introducing selection bias.

Scleral buckling remains a valuable technique, but limited exposure during training presents challenges for trainees. This study identifies procedural steps—especially binocular indirect ophthalmoscopy and scleral suturing—that are consistently time-consuming and less responsive to experience, highlighting areas for targeted education. These findings can help guide more efficient, competency-based training and support the preservation of SB proficiency among future retinal surgeons.

## Data Availability

No datasets were generated or analysed during the current study.
